# Oral administration of methysticin improves cognitive deficits in a mouse model of Alzheimer's disease

**DOI:** 10.1016/j.redox.2017.04.024

**Published:** 2017-04-19

**Authors:** Athanassios Fragoulis, Stephanie Siegl, Markus Fendt, Sandra Jansen, Ulf Soppa, Lars-Ove Brandenburg, Thomas Pufe, Joachim Weis, Christoph Jan Wruck

**Affiliations:** a Department of Anatomy and Cell Biology, Uniklinik RWTH Aachen University, Aachen, Germany; b Department of Pharmacology and Toxicology, Uniklinik RWTH Aachen University, Aachen, Germany; c Institute for Pharmacology and Toxicology, Medical Faculty, University of Magdeburg, Magdeburg, Germany; d Center of Behavioral Brain Sciences, University of Magdeburg, Magdeburg, Germany; e Institute of Neuropathology, Uniklinik RWTH Aachen and JARA Brain Translational Medicine, Aachen, Germany

**Keywords:** AD, Alzheimer's Disease, APP, amyloid beta precursor protein, ARE, antioxidant response element, Aβ, amyloid-beta, BCA, bicinchoninic acid, BSA, bovine serum albumin, GFAP, Glial fibrillary acidic protein, Iba1, ionized calcium-binding adapter molecule 1, IFNγ, interferon gamma, IL-1β, interleukin-1 beta, IL-10, interleukin-10, IL-17A, interleukin-17a, IL-6, interleukin-6, Nrf2, nuclear factor erythroid 2-related factor 2, PBS, phosphate buffered saline, Psen1, presenilin-1, qRT-PCR, quantitative reverse-transcriptase polymerase chain reaction, TNF-α, tumor necrosis factor-alpha, Methysticin, Kavalactone, Kava kava, Nrf2, Alzheimer's disease, Neuroinflammation, Astrogliosis, Oxidative stress

## Abstract

**Introduction:**

There is increasing evidence for the involvement of chronic inflammation and oxidative stress in the pathogenesis of Alzheimer's disease (AD). Nuclear factor erythroid 2-related factor 2 (Nrf2) is an anti-inflammatory transcription factor that regulates the oxidative stress defense. Our previous experiments demonstrated that kavalactones protect neuronal cells against Amyloid β (Aβ)-induced oxidative stress in vitro by Nrf2 pathway activation. Here, we tested an in vivo kavalactone treatment in a mouse model of AD.

**Methods:**

The kavalactone methysticin was administered once a week for a period of 6 months to 6 month old transgenic APP/Psen1 mice by oral gavage. Nrf2 pathway activation was measured by methysticin treatment of ARE-luciferase mice, by qPCR of Nrf2-target genes and immunohistochemical detection of Nrf2. Aβ burden was analyzed by CongoRed staining, immunofluorescent detection and ELISA. Neuroinflammation was assessed by immunohistochemical stainings for microglia and astrocytes. Pro-inflammatory cytokines in the hippocampus was determined by Luminex multi-plex assays. The hippocampal oxidative damage was detected by oxyblot technique and immunohistochemical staining against DT3 and 4-HNE. The cognitive ability of mice was evaluated using Morris water maze.

**Results:**

Methysticin treatment activated the Nrf2 pathway in the hippocampus and cortex of mice. The Aβ deposition in brains of methysticin-treated APP/Psen1 mice was not altered compared to untreated mice. However, methysticin treatment significantly reduced microgliosis, astrogliosis and secretion of the pro-inflammatory cytokines TNF-α and IL-17A. In addition, the oxidative damage of hippocampi from APP/Psen1 mice was reduced by methysticin treatment. Most importantly, methysticin treatment significantly attenuated the long-term memory decline of APP/Psen1 mice.

**Conclusion:**

In summary, these findings show that methysticin administration activates the Nrf2 pathway and reduces neuroinflammation, hippocampal oxidative damage and memory loss in a mouse model of AD. Therefore, kavalactones might be suitable candidates to serve as lead compounds for the development of a new class of neuroprotective drugs.

## Highlights

• Methysticin activates the Nrf2/ARE system in the hippocampus of mice.
• Methysticin protects AD mice against oxidative stress and associated neuroinflammation due to Nrf2 activation.
• Methysticin improves long-term memory impairment in this mouse model of AD.

## 1 Introduction

Alzheimer's disease (AD) is the most common form of dementia and is one of the most expensive diseases for industrial countries. It is clinically defined by progressive loss of cognitive and behavioral functions and is histopathologically characterized by amyloid beta deposits, neurofibrillary tangles, synaptic and neuronal loss. Additional features of AD are microglia activation and astrogliosis [1], [2].

Previously, we have shown that kavalactones attenuate amyloid ß (Aβ)-peptide toxicity by inducing protective gene expression mediated by the transcription factor nuclear factor erythroid 2-related factor 2 (Nrf2) in vitro [3]. Nrf2 activation regulates the transcription of phase-II detoxifying enzymes via the antioxidant response element (ARE) and, consequently, the redox homeostasis in numerous cell types, including glia cells and neurons. Up-regulation of Nrf2 activity renders neural cells more resistant to neurodegenerative diseases [4], [5], [6]. This is supported by the finding that haplotype variants of the Nrf2 gene NFE2L2 influence the progression of Alzheimer's disease [7]. Ramsey et al. showed that Nrf2 is localized in the cytosol rather than in the nucleus in both neurons and astrocytes in AD, even if Nrf2-activating oxidative stress and misfolded proteins are present [8].

The present study was designed to test the beneficial effects of the kavalactone methysticin on Aβ -toxicity in vivo. We used a transgenic mouse model of AD (APP/Psen1 mice). These mice co-express the mutated human amyloid beta precursor protein (APP) and mutated human presenilin 1 (Psen1) (B6.Cg-Tg(APP695)3Dbo Tg(PSEN1dE9)S9Dbo/J). Aβ aggregation is prominent in this mouse model. At 6 months of age, APP/Psen1 mice exhibit detectable amyloid plaque deposition, but do not show differences in cognitive function compared to their wild type littermates of the same age. Older double-transgenic mice exhibit impaired performance in all cognitive tasks, and the deficits correlate with total Aβ loads in the brain [9].

Kavalactones are extracted from the rhizome and roots of kava (Piper methysticum, G. Forst), a plant of the piperaceae family that is common on some Pacific islands [10]. Anthropological evidence suggests that kava has been cultivated and consumed for over 3000 years. It is still used today by a wide range of Pacific societies for spiritual, medicinal, and recreational purposes. Their psychopharmacological properties are already well known. The most commonly observed effects of low doses of kava extract are a relaxed mood or euphoric behavior, depending on the circumstances of ingestion. At higher doses, however, it causes sleepiness and skeletal muscle relaxation [11], [12]. Kavalactones have been shown to exhibit only moderate antioxidant activity [13], Several other effects have been reported, such as inhibition of nuclear factor-κB [14], iNOS [15] and cyclooxygenases [13], and neuroprotection after focal cerebral ischemia in mice and rats [16]. Furthermore, kava has no significant negative effects on cognition [17]. Yet, no interactions between kavalactones and neuroreceptors have been found that would explain the various pharmacological effects.

In our experiment we administered methysticin, one of the major kavalactones, to double-transgenic mice with prominent AD pathology by oral gavage. The treatment reduced memory deficits and neuropathological markers. In light of these result, kavalactones appear to be suitable candidates in the development of a new class of neuroprotective drugs.

## 2 Material and methods

### 2.1 Chemicals and preparation

Methysticin was obtained from LKT laboratories Inc. (Cat. #: M1679; St. Paul; MN, USA) and administered by oral gavage. Therefore methysticin was dissolved in DMSO at a concentration of 60 mg/mL and stored as stock solution at −20 °C. Before treatment the methysticin was further diluted in PBS to a final concentration of 1.5 mg/mL (1:40 dilution), resulting in a final DMSO concentration of 2.5%. The animals were treated with 6 mg/kg body weight (0.15 mg/25 g mouse, corresponding to 100 µL of working solution).

### 2.2 Animals

The animals that underwent behavioral testing and which were used for immunohistochemical and biochemical analyses were 52-week old adult mice of the B6.Cg-Tg(APP695)3Dbo Tg(PSEN1dE9)S9Dbo/J strain (APP/Psen1) as well as age-matched wild type littermates. The mice were purchased from The Jackson Laboratory (Jax^®^ Stock No.: 005866). Methysticin-mediated ARE/Nrf2 activation was investigated in transgenic C57BL6/J ARE-luciferase reporter gene mice purchased from Cgene (Oslo, Norway) [18]. All animals used in this study were housed in our animal facility under a 12 h light-dark cycle (lights on at 07:00 h), at regulated room temperature (22±2 °C), under specific pathogen-free conditions, and with ad libitum access to standard rodent diet and tap water. All experiments were conducted in accordance with the European animal protection directive 2010/63/EU and approved by regional governmental authorities (LANUV NRW 8.87-50.10.37.09.112).

### 2.3 Methysticin administration and sample preparation

Treatment of transgenic APP/Psen1 mice (n=6) was started at an age of 25 weeks and lasted for 27 weeks. The animals were treated once a week with 6 mg/kg bodyweight methysticin. Control groups consist of wild type mice (n=6) and APP/Psen1 mice (n=6) which were vehicle-treated with an identical treatment regimen. At 52 weeks, the animals underwent behavioral testing and were euthanized afterwards. The brain hemispheres were separated to obtain both formalin-fixed tissue for paraffin embedding and tissue for biochemical analysis from the same animal. Therefore, the left hemisphere was further dissected to separate the hippocampus from the remaining brain tissue. The fresh tissue was snap-frozen and immediately stored at −80 °C. To induce Nrf2/ARE in the ARE-luciferase reporter gene mice, the mice received 6 mg/kg bodyweight of methysticin once. The mice's hippocampus, cortex, midbrain, and cerebellum were prepared and immediately snap-frozen in liquid nitrogen 6 h after methysticin treatment.

### 2.4 ARE reporter gene assay

ARE-luciferase mice (n =6) were treated and tissue specimen prepared as described above (Section 2.3.). For the luminometer assay, the tissue was lysed in passive lysis buffer from Promega Corporation (Madison; WI, USA). Homogenization was intensified by mechanical force in a Precellys® homogenizer for 20 s at 5000 rpm to obtain whole protein homogenates. Luminescence measurement was conducted in a Glomax® 96 microplate luminometer with the Luciferase Assay Reagent I (LAR I), as recommended by the manufacturer. The signal was normalized against applied tissue mass.

### 2.5 qRT-PCR

Total RNA was extracted from hippocampus specimen by peqGOLD RNAPure™ (VWR Peqlab, Cat.#: 30-1030; Darmstadt, Germany) according to the manufacturer's recommendations. The nucleic acid concentration and purtity was determined with the NanoDrop® ND1000 device (Thermo Scientific, Wilmington, DE USA). RNA integrity was checked by MOPS buffered denaturizing RNA gel electrophoresis (rRNA ratio of 28S/18S of at least 1.8). 2 µg of total RNA was reverse transcribed as recommended by the supplier using the Maxima Reverse Transcriptase (Thermo Scientific, Wilmington, DE USA) with mixed priming strategy (oligo-(dT)18:random hexamer). Real-time PCR was conducted and monitored on an ABI StepOne Plus system using PowerSYBR® Green PCR Master Mix (Thermo Scientific, Wilmington, DE USA). All reactions were performed with primer-specific pre-evaluated annealing temperatures. Melt curve analysis and TAE-buffered DNA agarose gel electrophoresis was applied to ensure primer specificity. Amplification efficiencies were calculated post-run with LinRegPCR 2016.0 software (Heart Failure Research Center, Amsterdam, The Netherlands) as described by Ramaker et al. [19]. Prior to the actual study the most suitable normalization strategy was evaluated by GeNorm analysis (part of Biogazelle's qbase+2.6 software) with 8 representative samples and 8 possible reference genes. Based on that target expression levels were normalized to a reference gene index from the expression of actin-beta (Actb), beta-1 microglobulin (B2m), glyceraldehyde 3-phosphate dehydrogenase (Gapdh), and hypoxanthine guanine phosphoribosyl transferase (Hprt). The relative fold- change of gene expression was calculated with qbase+2.6 software (Biogazelle, Gent, Belgium). All applied primer pairs are listed in Table 1.

**Table 1:** Primer sequences and applied annealing temperatures in qRT-PCR reactions.

**name**	**forward**	**reverse**	**Annealing**
Actb	CAC TGT CGA GTC GCG TCC	TCA TCC ATG GCG AAC TGG TG	60,0 °C
B2m	TTC TGG TGC TTG TCT CAC TGA	CAG TAT GTT CGG CTT CCC ATT C	61,0 °C
Gapdh	CAT GGC CTT CCG TGT TCC TA	ACT TGG CAG GTT TCT CCA GG	60,0 °C
Hprt	TCA GTC AAC GGG GGA CAT AAA	GGG GCT GTA CTG CTT AAC CAG	61,0 °C
Ho-1	AAG CCG AGA ATG CTG AGT TCA	GCC GTG TAG ATA TGG TAC AAG GA	61,5 °C
Gclc	GGG GTG ACG AGG TGG AGT A	GTT GGG GTT TGT CCT CTC CC	60,5 °C

### 2.6 Behavioral tests

The mice were kept in their home cages in the laboratory for 30 min prior to all behavioral tests so that they were able to adapt to their new environment. The temperature was held stable at 22±2 °C, and the room was slightly illuminated.

#### 2.6.1 Open-Field

The mice's locomotion, anxiety, and exploratory behavior were investigated using an open field apparatus (W×H×D: 50 cm×50 cm×50 cm). All animals underwent this test once. For this purpose, they were placed in the center of the maze and given ten minutes to freely explore it. The animals were tracked with a Stoelting Co. ANY-maze™ video tracking system and software (Wood Dale, Illinois, USA); data were collected concerning mean speed, relative time spent in different zones of the maze, and the total distance each animal traveled during the experiment.

#### 2.6.2 Y-Maze

The apparatus used to test the animals’ working memory was an Y-Maze with three identical arms (W×H×D of each arm: 9 cm×16 cm×40 cm) arrayed at 120° angles. The floor and walls were painted white to provide maximal contrast to the animals’ color. All animals underwent this test once. To begin the test, the animals were placed in the lower arm of the maze (C) and given six minutes to freely explore it. Each mouse's mean speed, total distance traveled, and the sequence of visited arms were monitored and analyzed with Stoelting Co. ANY-maze™ video tracking system and software (Wood Dale, Illinois, USA). For statistical analysis, the spontaneous alternation (SA%) of arm visits was calculated as depicted in Eq. (1). *Totalvisits* is the sum of all visited arms. Alternations defines the amount of events when the animal chose a different arm than that it was coming from when leaving the actual arm (e.g. arm sequence: ABC → first arm was A, then the animal entered arm B and left it choosing arm C instead of revisiting arm A).

(1) SA%=[(totalvisits−2)÷alterations]×100.

#### 2.6.3 Morris water maze

To assess the animals’ long-term memory performance, we used a circular Morris water maze (diameter 120 cm; height 50 cm and a water temperature of 24 °C). The maze was filled with white-stained water to provide maximal contrast to the animals’ color. The maze was divided into four quadrants, equipped with 4 landmarks at the inside of the wall. The white escape platform (diameter 10 cm; height 24 cm) was located 1 cm below the water surface. The animals were subjected to the maze each day for a time period of 5 days, with 6 trials per day (3 trials in the morning and 3 trials in the afternoon). The three contiguous trials were held at 5 min intervals, and the morning and afternoon sessions were separated by 5-h interval. The experiment was divided into three stages: flagged trials (days 1 and 2, trials 1-12), training trials (days 3 and 4, trials 1-12), and probe trial (day 5, trial 1). For the flagged trials the platform position was clearly indicated by a black flag and all other cues were removed from the maze. During these flagged trials the platform was located at 4 variable positions and the animals were inserted at a constant position. For the training sessions, the flag was removed from the platform and the animals were inserted at different starting points with a constant platform position. In the probe trial, the animals were inserted in the upper left quarter and the platform was placed in the bottom right quarter of the maze. In each trial mice were placed at one of the distinct places, faced to the wall, and were allowed to swim freely until they reached the hidden platform. Mice, which failed to find the platform within 60 s, were placed on the platform for 10 s (equal time as successful animals stayed on it). One floating wild type mouse was excluded at the beginning of the MWM experiment and replaced by another wild type mouse. ANY-maze™ was used to track the animals during the test and to obtain data regarding their latency, the corrected integrated path length, average distance from the platform, path efficiency, average swimming speed, and covered distance. Moreover, the software was used to generate track plots for each animal. These data were used to analyze the mice's long-term memory and their learning curves.

### 2.7 Histology and Immunohistochemistry

All mice (n=6 per group), which underwent behavioral testing were sacrificed at the age of 52 weeks. Their brains were removed and dissected median-sagittal. The left hemisphere was fixed in 4% neutral buffered formalin for 24 h and embedded in paraffin afterwards. The Aβ load in the brain tissue was investigated by Congo Red and immunofluorescent staining with a specific antibody against Aβ1-42 (Millipore: Cat.# AB5078P, Darmstadt, Germany) of 5 µm-thick sections. The Amyloid stain Congo Red kit was purchased from Sigma Aldrich (Cat. #: HT-60-1KT; Munich, Germany) and used according to the manufacturer's instructions. For immunohistochemical analysis the paraffin-embedded samples were deparaffinized in xylene and rehydrated in dilutions of ethanol and deionized water. Antigens were retrieved by heating sections in antigen retrieval solution (15 mL of 1 M sodium citrate and 15 mL of 1 M citric acid in deionized water, pH 6.0) in a microwave for 20 min. Slides were washed in phosphate buffered saline (PBS) and 0.05% Tween for 5 min. Samples were treated with 5% bovine serum albumin (BSA) in PBS for 30 min to block non-specific binding. Antibodies against glial fibrillary acidic protein GFAP 1:10000 (EnCor Biotechnologies Inc.: Cat.# RPCA-GFAP; Gainesville, FL, USA), ionized calcium binding adapter molecule 1 Iba1 1:10000 (Wako Chemicals: Cat.# 019-19741; Neuss, Germany), amyloid-beta Aβ1-42 1:150, dityrosine DT3 1:7500 (Jaica, Cat.# MDT-020P; Fukuroi, Japan), 4-hydroynonenal 4-HNE 1:100 (abcam, Cat.#: ab48506; Cambridge, UK), and nuclear factor E2-related factor 2 Nrf2 1:300 (GeneTex, Cat.#: GTX103322; Irvine, CA USA) were used to visualize astrogliosis, microglia infiltration, Aβ deposition, oxidative stress-associated damage and Nrf2 localization. The first antibody was omitted in No-Primary controls. Slides were incubated with 3% hydrogen peroxide for 10 min at room temperature to block endogenous peroxidase activity. Slides were washed in PBS and incubated with secondary antibody conjugated with HRP (Dako, Hamburg, Germany) for GFAP, Iba1, DT3, 4-HNE and Nrf2 detection or with Alexa488 (life technologies: Cat.# A21206, Darmstadt, Germany) for Aβ visualization at room temperature for 1 h. AEC (3-Amino-9-ethylcarbazole) or DAB (3,3′-diaminobenzidine) were used as recommended by the manufacturer (Life Technologies, Darmstadt, Germany) to visualize permanent stainings of GFAP, Iba1, DT3, 4-HNE and Nrf2. 1:3 Mayer's hematoxylin (BDH Laboratories, Poole, UK) was incubated for 30 s as a DNA counter stain prior to mounting in Kaiser's glycerol. For the immunofluorescent stainings DAPI was used as DNA counter stain, respectively. The sections were analyzed with a Keyence BZ-9000 microscope using the MERGE function on a 20x objective. The immunoreactivity of 3 consecutive sections of selected stainings (Iba1 and GFAP) was quantified with the BZ-Analyzer measurement module “color extraction mode”. To do so, the positive stained area was measured and related to the total area of each hippocampus. This data was then normalized to the WT group to depict “x-fold” immunoreactivity of WT.

### 2.8 Oxyblot

Protein oxidation was shown by determining levels of protein carbonylation. By this method carbonyls are derivatizatized with 2,4-dinitrophenylhydrazine (DNPH), that can be detected with a specific anti-DNP antibody on immunoblots.[20] Hippocampal samples (5 µL containing 10 µg total protein) were initially denaturated by adding 5 µL of 12% SDS and boiling at 95 °C for 15 min. Derivatization was achieved subsequently by the addition of 5 µL 10 mM DNPH (in 2 N HCl) solution and incubation at room temperature (RT) for 20 min. Samples were subsequently neutralized with 7.5 µL of neutralization solution (2 M Tris in 30% glycerol) and loaded on 12% polyacrylamid gels for electrophoretic separation. For controls, the same samples were treated as described except that a control solution (2 N HCl without DNPH) was used instead of the DNPH-containing derivatization solution. Proteins were blotted to a PVDF membrane. Washing steps were done with TBS solution containing 0.04% (v/v) Tween 20 (WB). Membranes were blocked at RT for 1 h with 3% bovine serum albumin dissolved in WB before incubation with the rabbit polyclonal anti-DNP primary antibody (1:100; cat.#: D9656; Sigma Aldrich, Frankfurt, Germany) at RT for 2 h under constant rocking. Immunoreactivity was visualized using the chemiluminescence reagent Immobilon™ Western (Millipore, Darmstadt, Germany) as recommended by the manufacturer. Luminescence signals were detected on photo-sensitive Hyperfilm™ ECL (GE Healthcare Life Science, Chalfont St. Giles; United Kingdom) in a dark room under exclusion of light. For densitometry the density of the DNP signals were captured using a flatbed scanner and the intensity was calculated with Quantity One Software 4.6.9 (Biorad; Hercules, CA USA). Equal loading was confirmed by AmidoBlack staining. After DNP detection the membrane was incubated in 0.1% AmidoBlack staining solution (solved in 45% methanol and 15% acetic acid) for 5 min. Destaining was conducted until the background was white again using AmidoBlack destaining solution (90% methanol and 2% acetic acid) with consistent changes of the solution every 5 min. The ratio between DNP immunoreactivity and the corresponding AmidoBlack stainings are represented graphically.

### 2.9 Aβ ELISA

Total Aβ content in the hippocampal tissue was quantified by Amyloid-beta (1-x) ELISA (IBL International; Cat. #: JP27729; Hamburg, Germany). The tissue was homogenized with the Bio-Plex Cell Lysis Kit (Bio-Rad laboratories; Cat. #: 171-304011; Munich, Germany) as recommended for tissue samples. Therefore 30 mg of hippocampal tissue was transferred to 2 mL Precellys tubes filled with 1.4 mm ceramic beads and 350 µL Cell Lysis Buffer. Homogenization was performed with the Precellys® 24 system. The protein content was determined by BCA assay and the samples were adjusted to 15 µg protein per 100 µL. The ELISA was conducted as recommended by the manufacturer. Resulting Aβ concentrations (pg/mL) were converted to pg/µg total protein.

### 2.10 Multi-plex luminex assay

Cytokine expression in the hippocampal tissue was assessed by Multi-Plex Luminex technology. The cytokines tumor necrosis factor-alpha (TNF-α), interleukin-17A (IL-17A), interleukin-1beta (IL-1β), interleukin-6 (IL-6), interferon gamma (IFNγ) and interleukin-10 (IL-10) were detected using the Bio-Plex Pro™ Mouse Cytokine Th17 Panel A 6-Plex Group l Kit (Bio-Rad laboratories; Cat. #: M60-00007NY; Munich, Germany) on a Luminex 200 system. For these analyses snap-frozen tissue of the hippocampus was used. The tissue was homogenized using the Bio-Plex® Cell Lysis kit (Bio-Rad laboratories; Cat. #: 171304011; Munich, Germany) as recommended by the company. The protein content was determined by BCA assay (Thermo Scientific, Pierce Protein Biology Products; Cat. #: 23227; Rockford, IL USA) and the samples were adjusted to 125 µg protein per 50 µL. The assay was conducted as recommended by the manufacturer. Resulting cytokine concentrations (pg/mL) were converted to pg/100 µg total protein.

### 2.11 Statistics

All data are presented as mean+SEM. Normal distribution was validated using the Shapiro-Wilk test. The Bartlett test was used to check for equal variances. Parametric data and mean comparisons of two groups were analyzed by Student's *t*-test. In the case of multiple comparisons, data were analyzed by one-way analysis of variance (ANOVA) followed by Tukey's multiple comparisons post-hoc test. Learning curves were analyzed by two-way repeated-measure ANOVA and Tukey's multiple comparisons post-hoc test. Percentage data were transformed using the arcsine transformation before statistical testing. All analyses were carried out with GraphPad Prism 5 Software (GraphPad; La Jolla, CA USA) and JMP 10 software (SAS; Cary, NC USA).

## 3 Results

### 3.1 Oral administration of methysticin activates the Nrf2 pathway in hippocampus and cortex

**Fig. 1:**
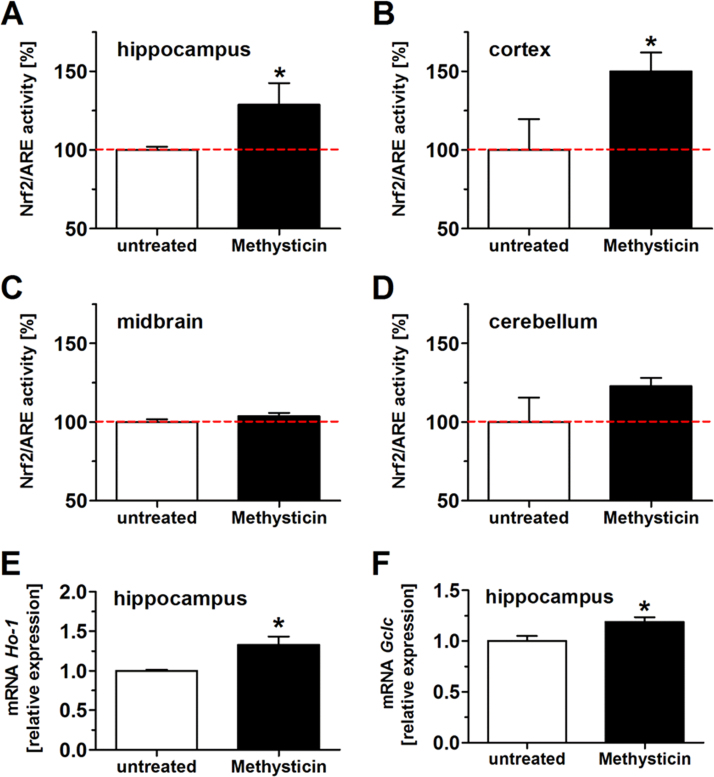
Methysticin application induced Nrf2 in the hippocampus and cortex of ARE-luciferase reporter gene mice. Nrf2 activation due to methysticin application was investigated in ARE-luciferase reporter gene mice. Animals were treated with methysticin once by oral gavage. Brains were prepared as described in the method section. Hippocampus, cortex, midbrain and cerebellum were separated and homogenized. The homogenates were used in a luminometer assay and for qRT-PCR. Methysticin significantly induced Nrf2/ARE activity in the hippocampus (A) and cortex (B) of treated animals. There was no effect on Nrf2/ARE activation in midbrain (C) and cerebellum (D). mRNA expressions of the Nrf2 target genes Ho-1 (E) and Gclc (F) were significantly increased after methysticin treatment (2-tailed unpaired *t*-test). Data represent mean+SEM; n =6; * p<0.05 vs. untreated.

Methysticin treatment resulted in a significant activation of the Nrf2/ARE pathway in hippocampus (Fig. 1A) and the cortex (Fig. 1B) but not in the midbrain (Fig. 1C) and cerebellum (Fig. 1D) of ARE-luciferase reporter gene mice. This was also confirmed by qRT-PCR analysis of the gene expression of the well-known Nrf2 target genes heme oxygenase-1 (Fig. 1E: Ho-1) and glutamate-cysteine ligase catalytic subunit (Fig. 1F: Gclc) in the hippocampus. Methysticin treatment significantly increased the expression of both genes compared to untreated animals.

**Fig. 2:**
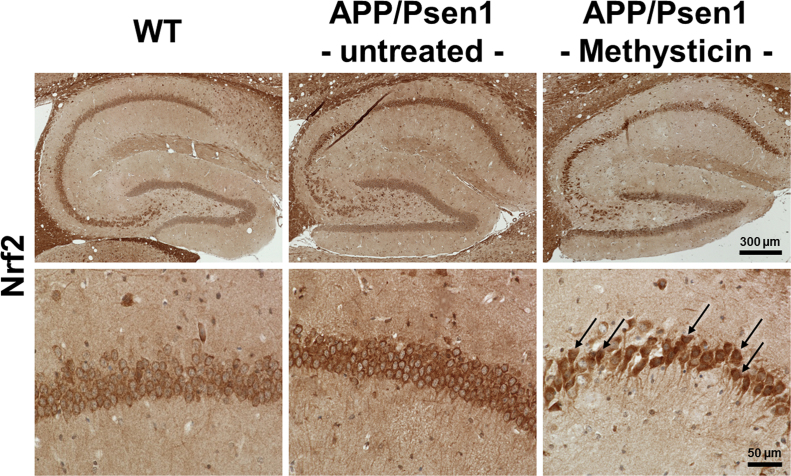
Methysticin induces Nrf2 nuclear translocation in hippocampal neurons. Nrf2 activity and its cellular localization in the hippocampus was determined by IHC staining. Nrf2 was predominantly found in the cytosol of hippocampal neurons in WT and untreated APP/Psen1 mice. Methysticin treatment led to nuclear translocation of Nrf2 and thereby induced its signaling. Overview of hippocampi (upper row) and detailed depiction of CA1 region (lower row) of representative stainings are shown. n=6.

We next studied Nrf2 activation in WT, untreated and methysticin treated APP/Psen1 mice in more detail. We performed stainings of hippocampal slices with antibody against Nrf2. Since Nrf2 is a transcription factor, activated Nrf2 is located within the nucleus. As seen in the study of Ramsey and colleagues, who demonstrated that Nrf2 was predominantly located in the cytoplasm of neurons from AD-patient [8], we observed an equal cellular localization of Nrf2 in our AD model. In both WT (Fig. 2, images on the left) as well as in APP/Psen1 mice (Fig. 2, images in the middle) the Nrf2 staining was predominantly found in the cytosol of the neurons, omitting the nucleus. Interestingly, methysticin treated APP/Psen1 mice showed Nrf2 staining in both the cytosol and the nucleus of the neurons (Fig. 2, images on the right).

### 3.2 Oral administration of methysticin reduces cognitive impairments in APP/Psen1 mice

The administration of methysticin affected neither the physical condition nor species-specific behavior of the animals, since neither the methysticin-treated nor the untreated APP/Psen1 mice differed from age-matched wild-type control animals in the open field maze. All three groups spent approximately 50% of the total time in corners, 40% at the sides, and only 10% in the center of the maze (Suppl. Fig. S1B; two-way ANOVA: genotype p=0.9804, zone p<0.0001, interaction p=0.2766), which is within the normal range. The mice did not differ with respect to their physical condition; all animals traveled approximately 20–30 m during the experiment, with a mean speed of 0.04–0.05 m/s. Animals treated with methysticin exhibited slightly reduced movement, but the reduction was not statistically significant (Suppl. Fig. S1C & D both p>0.05). The working memory was neither affected in untreated nor in the treated APP/Psen1 mice, when compared to the control mice in the Y-maze evaluation. All three groups exhibited a spontaneous alternation (%SA) of 50–60% (Suppl. Fig. S2B). The traveled distances were reasonably equal as well, with 15–20 m (Suppl. Fig. S2C) and a mean speed of approx. 0.05 m/s (Suppl. Fig. S2D).

**Fig. 3:**
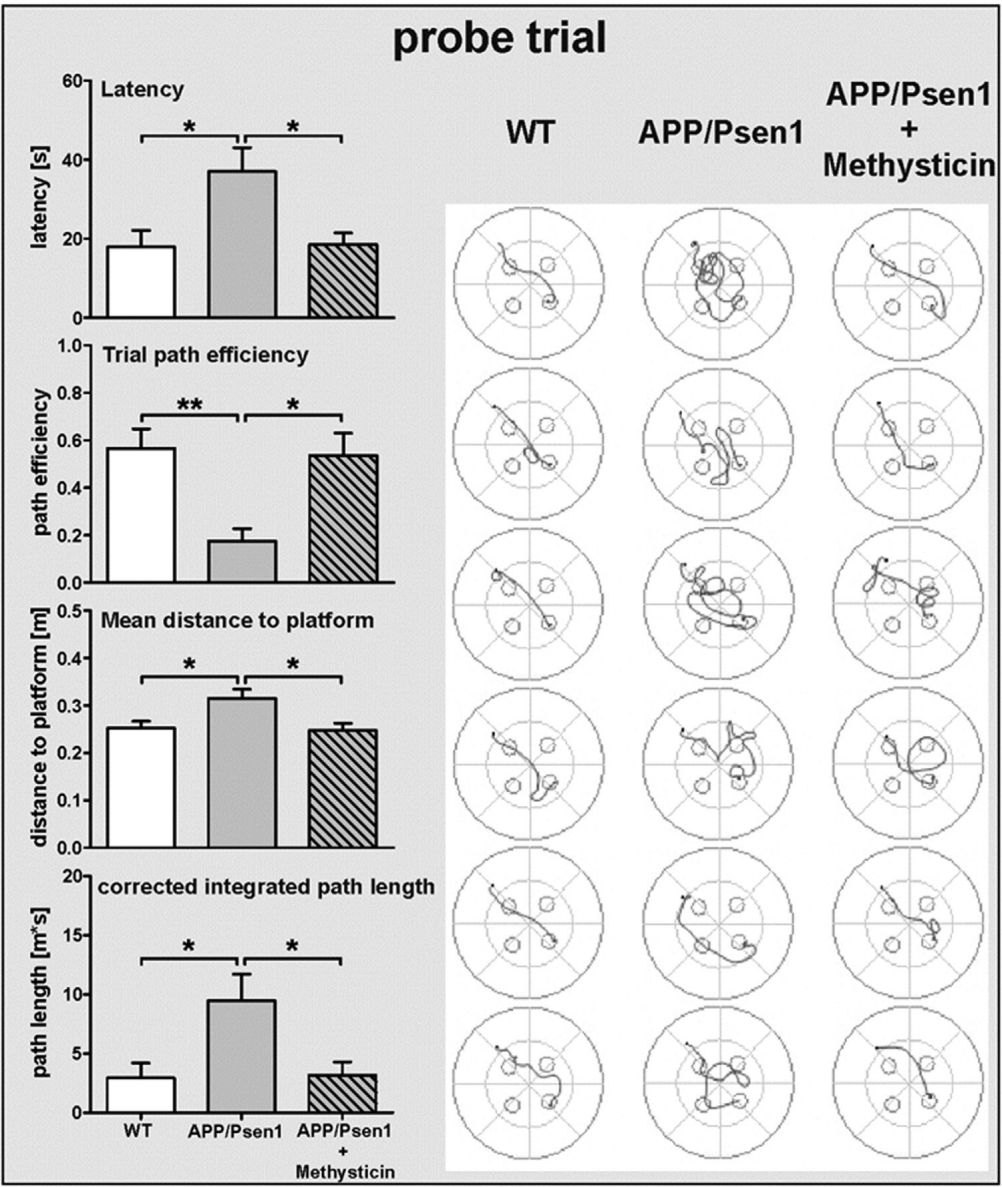
Schematic illustration of the Morris-Water Maze experimental design. To analyze long-term memory we performed a Morris water maze test. The apparatus measured 120 cm in diameter. To test long-term memory, latency, path efficiency, mean distance to the platform and the corrected integrated path length during the probe trial were analyzed. Track plot of each individual animal from the probe trial on day 5 are shown. APP/Psen1 mice showed a significant decline in long-term memory performance compared to age-matched wild-type littermates. Methysticin treatment rescued this effect significantly. (Statistics probe trial: one-way ANOVAs with Tukey's multiple comparisons post hoc test). Data represent mean+SEM; n=6; * p<0.05, ** p<0.01 as indicated.

However, the Morris water maze test demonstrated that all three groups were comparable with respect to learning ability but not with respect to their long-term memory performance. Quality control data regarding locomotive abilities (Suppl. Fig. S3, left panel: traveled distance and swim speed) of the animals were assessed during the flagged trials and show no statistical difference between all groups. Statistical analyses of learning curves during the training sessions revealed a significant trial effect in the course of training without significant influence of the genotype (Suppl. Fig. S3, right panel). Regarding the access to the acquired long-term memory in the probe trial, the untreated APP/Psen1 mice were severely impaired compared to their age-matched wild type littermates (see track plots in Fig. 3): latency (28.3 s vs. 18.0 s), mean distance to the platform (0.33 m vs. 0.24 m) as well as corrected integrated path length (9.83 m*s vs. 2.94 m*s) were significantly increased (Fig. 3, gray bars vs. white bars). Additionally, path efficiency was decreased in untreated APP/Psen1 (0.14 vs. 0.56). Methysticin treatment rescued this long-term memory decline. The methysticin-treated animals found the platform within 19.7 s with a corrected integrated path length of 2.35 m*s, a mean distance to the platform of 0.23 m and a path efficiency of 0.57 (Fig. 3, right panel: gray patterned bar).

### 3.3 Oral administration of methysticin does not affect Aβ deposition in APP/Psen1 mice

**Fig. 4:**
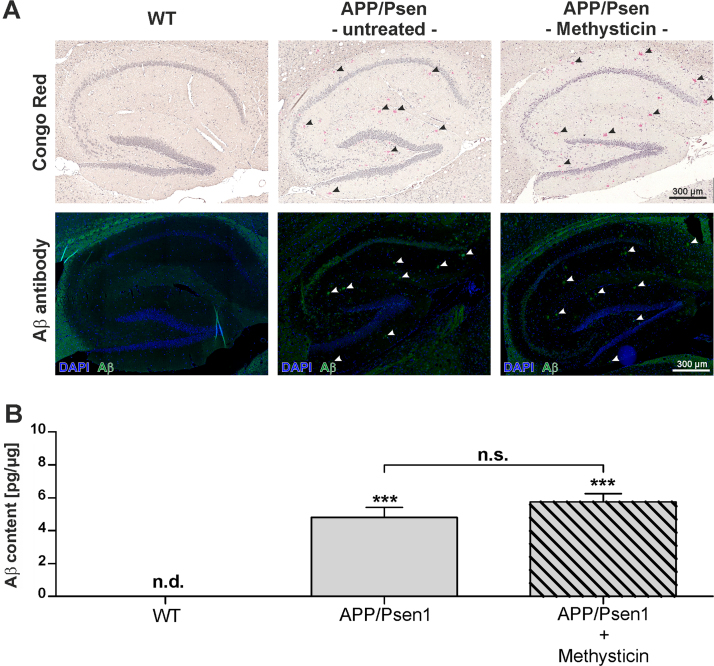
Methysticin treatment did not significantly prevent amyloid-beta deposition in APP/Psen1 hippocampus. To visualize and quantify amyloid-beta deposition, Congo Red and immunohistochemical staining with a specific antibody against Aβ1-42 were conducted. The analyses were focused on the hippocampus. (A) Amyloid-beta plaque density in this area was analyzed by scanning the hippocampus at 20× magnification. Single pictures were merged using the BZ-II Analyzer Software (Keyence, Neu-Isenburg, Germany). Representative pictures (n=6) of wild type (1st column), untreated APP/Psen1 (2nd column) and methysticin treated (3rd column) hippocampi are shown. The first row represents the pictures of Congo Red staining and the second row the immunohistochemical staining against Aβ1-42. (B) Aβ content in the hippocampus of investigated animals was determined by ELISA. Hippocampal tissue was processed as described in the methods section and data are expressed as pg/µg total protein. (one-way ANOVA with Tukey's post hoc test). Data represent mean+SEM; n=6; *** p<0.001 vs. WT, n.d. not detectable.

Microscopic scans of the whole sagittal section of the APP/Psen1 mice revealed Aβ deposition in all areas (data not shown). The Aβ plaque load in the hippocampi was analyzed by staining with Congo Red and an antibody against Aβ1-42 (Fig. 4A). Aβ was only detected in methysticin treated as well as untreated APP/Psen1 mice. However, there was no significant difference in the plaque load between untreated and methysticin treated mice. The detection of total Aβ by ELISA technique further confirmed that finding (Fig. 4B).

### 3.4 Oral administration of methysticin decreases astrogliosis and microglia activation

**Fig. 5:**
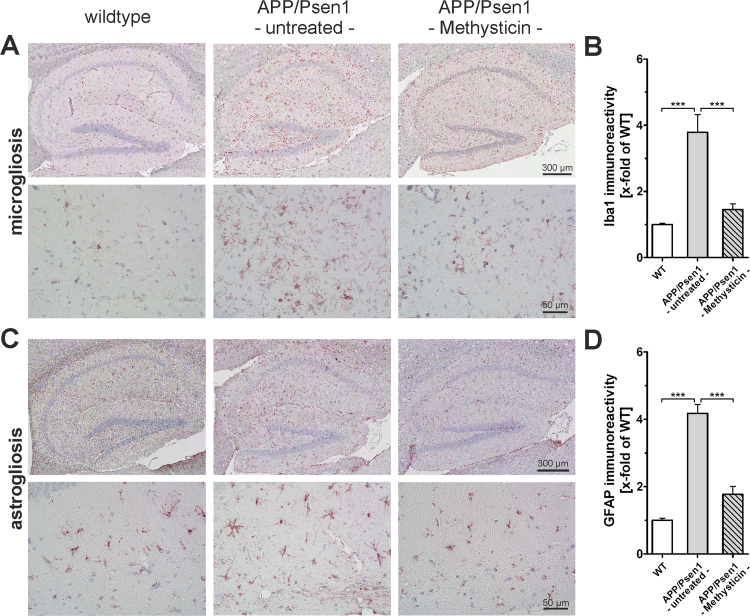
Neuroinflammation in hippocampi of APP/Psen1 mice is reduced by methysticin treatment. (A & C) Microglia infiltration and astrogliosis was analyzed by immunohistochemical staining against Iba1and GFAP respectively (n=6). Pictures were generated by scanning the hippocampus at 20× magnification and subsequent merging of these pictures to a single picture. The upper lanes in A and C show representative pictures of wild type (1st column), untreated APP/Psen1 (2nd column) and methysticin-treated APP/Psen1 (3rd column) mouse hippocampi. The lower lanes in A and C provide detailed pictures at 40× magnification of the same groups. Scale bars: upper lanes=300 µm, lower lanes =50 µm. (B & D) Quantification of immunoreactivity of 3 consecutive sections of each hippocampus. Untreated APP/Psen1 mice showed prominent microglia infiltration and astrogliosis that was extensively elevated compared to wild type (WT) mice. Methysticin application led to a significant reduction of microglia infiltration as well as astrogliosis in these animals. (one-way ANOVA with Tukey's multiple comparison post hoc test). Data represent mean+SEM; n=6; *** p<0.001 as indicated in the chart.

Reactive gliosis in the meaning of microglia and astrocytes activation was investigated by immunohistochemistry with antibodies against Iba1 (Fig. 5A and B: microglia) and GFAP (Fig. 5C and D: astrocytes). 52 weeks old APP/Psen1 mice showed both an increased microglia cell and astrocyte population in the hippocampus when compared to age-matched wild type animals (Fig. 5B & D, 1st and 2nd columns). Methysticin treatment of APP/Psen1 mice led to a significant reduction of Iba1 and GFAP staining intensity when compared to untreated APP/Psen1 mice (Fig. 5B & D, 3rd column).

**Fig. 6:**
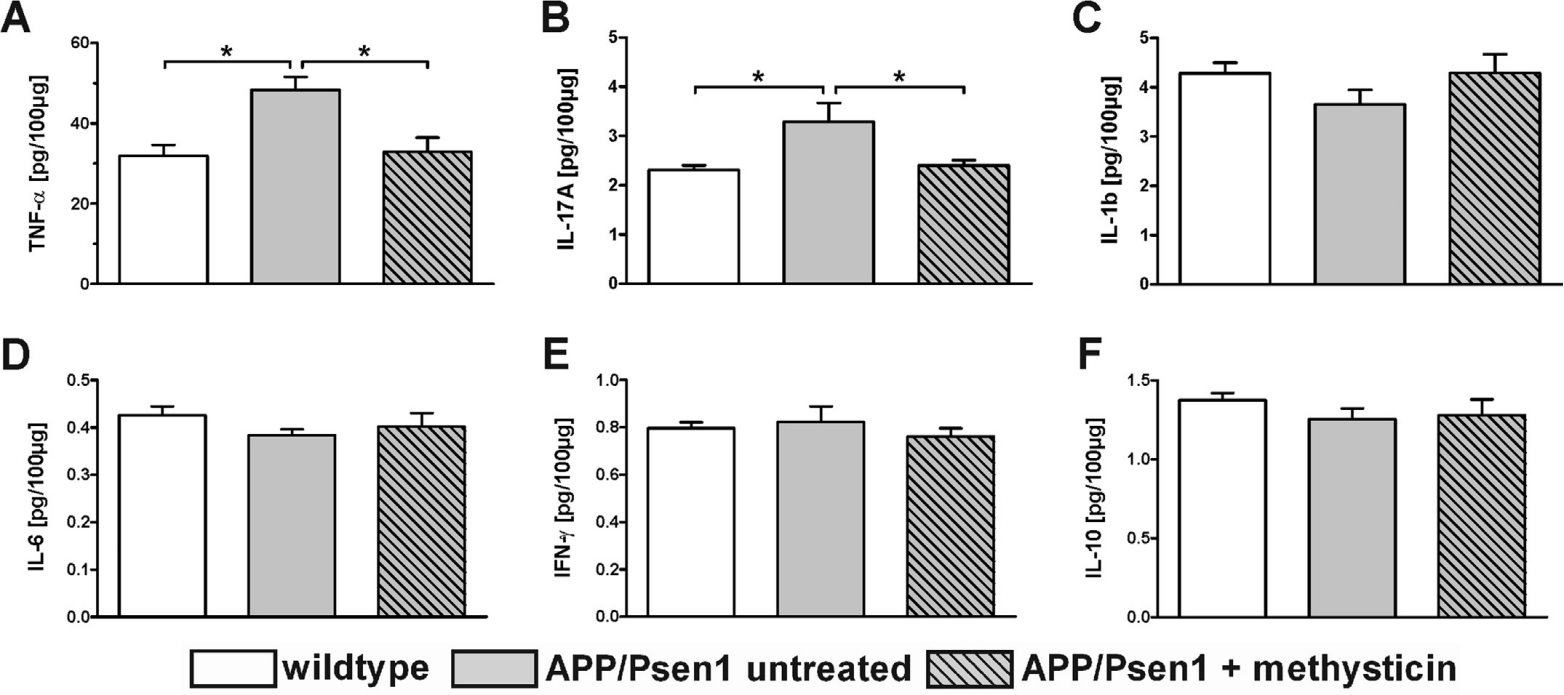
The secretion of pro-inflammatory cytokines in hippocampi of APP/Psen1 mice is reduced by methysticin treatment. Cytokine secretion in the hippocampus was analyzed by Luminex Multi-plex assay. Hippocampal tissue was processed and the assay was conducted as described in the methods section. Cytokine secretion is expressed as pg/100 µg total protein. The pro-inflammatory cytokines TNF-α (A) and IL-17A (B) were significantly increased in untreated APP/Psen1 animals compared to the age-matched wild type control animals. Methysticin treatment in turn prevented this effect. The other investigated inflammatory mediators (IL-1b, IL-6, IFN-γ and IL-10) did not differ between the groups (C – F). (one-way ANOVA with Tukey's multiple comparison post hoc test). Data represent mean+SEM; n=6; * p<0.05 as indicated.

Cytokine concentration was detected in the hippocampus of animals via Luminex multi-plex technique (Fig. 6). This analysis showed a significantly increased expression of the pro-inflammatory mediators TNF-α (Fig. 6A) and IL-17A (Fig. 6B) in untreated APP/Psen1 animals compared to age-matched wild type mice (TNF-α: 48.35 vs. 31.85 pg/100 µg protein, IL-17A: 3.29 vs. 2.30 pg/100 µg protein). Methysticin treatment significantly reduced the secretion of these cytokines to wild type levels (TNF-α: 32.87 pg/100 µg protein, IL-17A: 2.40 pg/100 µg protein). The other investigated cytokines IL-1b, IL-6, IFN-γ and IL-10 (Fig. 6C-F) did not differ between the groups.

### 3.5 Oral administration of methysticin decreases oxidative damage of the hippocampus of APP/Psen1 mice

**Fig. 7:**
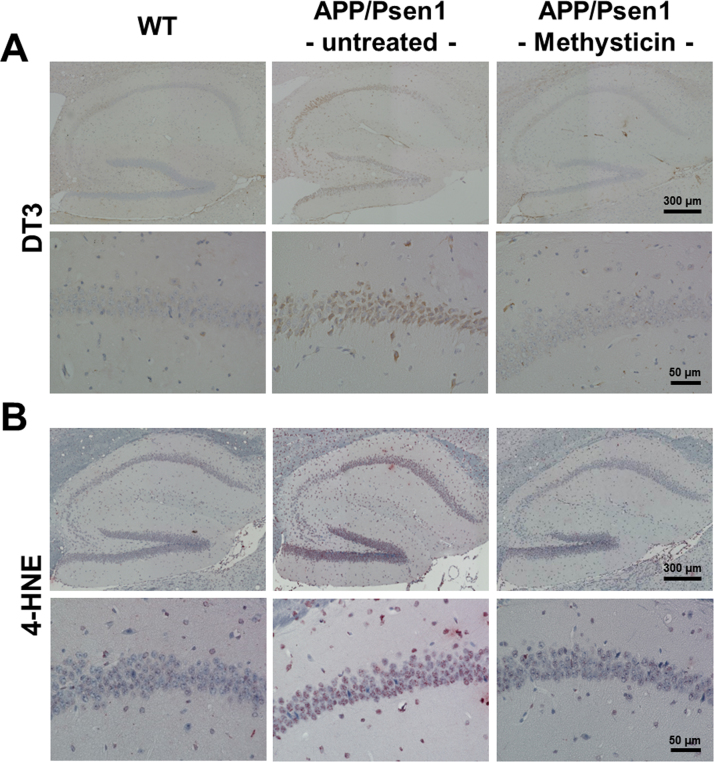
Methysticin reduces oxidative stress mediated damage. Oxidative damage to amino acids was analyzed by immunohistochemistry using antibodies against DT3 (A) and 4-HNE (B). The elevated oxidative protein modification induced by the APP/Psen1-transgene was significantly reduced by methysticin treatment. n=6.

**Fig. 8:**
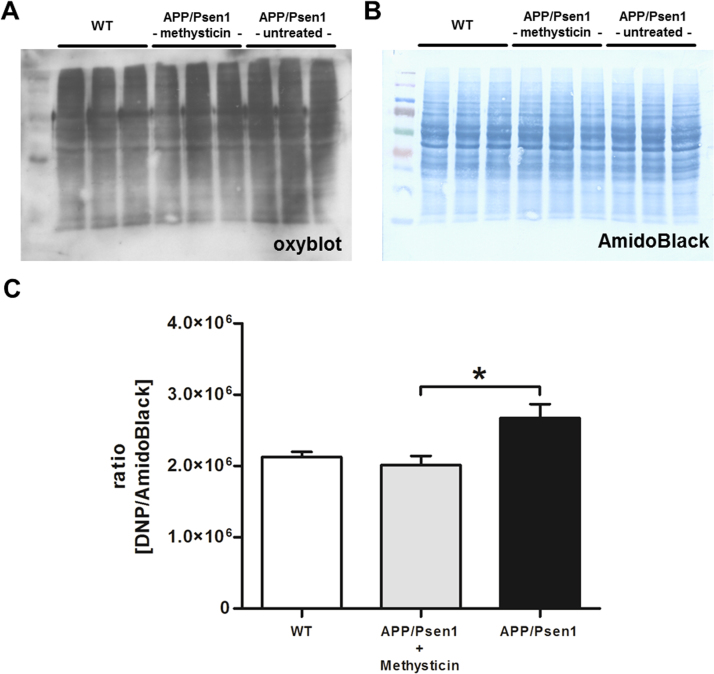
Methysticin reduces oxidative stress mediated damage. Oxidative damage to amino acids was additionally analyzed by oxyblot technique. The oxyblot using an DNP-specific antibody (A) and the corresponding loading control by AmidoBlack staining (B) is shown. Densitometry revealed increased protein carbonylation in untreated APP/Psen1 mice compared to WT littermates. Methysticin treatment reduced protein carbonylation to WT levels. (one-way ANOVA with Tukey's multiple comparison post hoc test). Data represent mean+SEM; n=6; * p<0.05 as indicated.

Nrf2 is the major regulator of the cellular defense machinery against oxidative stress. We therefore studied the amount of oxidative damage induced by APP/Psen1-expression in APP/Psen1 mice. First, we detected oxidized amino acid residues in protein with antibodies against dityrosine- (DT3) and 4-hydroxynonenal- (4-HNE) adducts in hippocampal slides. The elevated oxidative protein modification induced by the APP/Psen1-transgene was significantly reduced by methysticin treatment (Fig. 7A and B). Moreover, the oxidative modification of hippocampal proteins was furthermore verified by Oxyblot assay. The densitometrical analysis of the oxyblot clearly demonstrated that the oxidative protein modification induced by APP/Psen1-expression was reduced by methysticin treatment (Fig. 8A-C).

## 4 Discussion

The present study demonstrates that methysticin, a natural occurring kavalactone, reduces long-term memory decline in middle-aged APP/Psen1 mice when administered orally. Although the Aβ deposition in the brains of methysticin-treated APP/Psen1 mice was not reduced, oxidative damage, microglial activation, astrogliosis and the secretion of the pro-inflammatory mediators TNF-α as well as IL-17A were significantly reduced in the methysticin treated APP/Psen1 mice as compared to the untreated APP/Psen1 mice.

Previously, our group showed that kavalactone-mediated Nrf2 activation protects against Aβ-toxicity in cultured neurons in vitro [3]. Further evidence that Nrf2 activation is neuroprotective against Aβ-toxicity was provided by Kanninen et al., whose elegant genetic approach demonstrated that viral transduction of Nrf2 into the hippocampus of APP/Psen1 mice improves their spatial memory [21], [22]. These studies proved the conceptual validity of Nrf2-activation approaches to Alzheimer's disease, but did not provide a practically feasible therapeutic approach for a widespread disease like AD.

The outcomes of our behavioral and histopathological analyses of APP/Psen1 mice treated with methysticin were comparable to these findings from the study of viral Nrf2 transduction [22]. Methysticin treatment significantly reduced spatial memory deficits in middle-aged APP/Psen1 mice in a manner similar to that described in this study. The observed effect did not result from increased Aβ clearance, because neither the total amount of Aβ nor the formation of Aβ plagues in the hippocampus were reduced. Nevertheless the treatment with methysticin reduced Aβ-induced astrogliosis, microglia infiltration and the secretion of pro-inflammatory cytokines, much like the effects observed in mice treated with Nrf2-expressing viruses. From the analogous character of these two studies’ outcomes and from our earlier findings [3], it stands to reason that the beneficial effect of methysticin on APP/Psen1 mice is the result of methysticin driven Nrf2 activation. We showed that oral administration of methysticin induced Nrf2 activity in the hippocampal and cortical areas of ARE-luciferase reporter gene mice. As both regions and especially the hippocampus play crucial roles for spatial learning and memory, this effect is in line with our hypothesis of Nrf2-dependent improvement of long-term memory performance. These results further confirm our findings from the previous in vitro study, in which kavalactones protected neuronal PC12 cells against Aβ induced cytotoxicity due to Nrf2 activation [3].

Neuroinflammation plays important roles in the pathophysiology of AD. It may contribute to neuronal dysfunction and cell death, like a self-perpetuating cycle by which inflammation feeds further neurodegeneration. In prospective studies, anti-inflammatory treatments have delayed AD onset and alleviated or slowed cognitive decline [23]. Therefore, an anti-inflammatory treatment may represent an appropriate therapy for AD. Unlike the virus-based method of inducing Nrf2, however, methysticin treatment significantly reduced microglial infiltration, astrogliosis and the secretion of the pro-inflammatory cytokines TNF-α and IL-17A in APP/Psen1 mice. Both cytokines are known to be present at higher levels in AD and are responsible for the concomitant neuroinflammation [24]. While TNF-α enhances migration of leukocytes in the inflamed region, increased IL-17A which is produced by Th17 cells indicates an enhanced Th17 pro-inflammatory response [25]. Literature gives evidence, that endothelial cells of the blood brain barrier (BBB) express IL-17 receptors and that binding of IL-17 to these receptors leads to the disruption of the BBB tight junctions [26]. This process facilitates further infiltration of leukocytes and Th17 cells into the brain, further amplifying the neuroinflammatory process [26]. Our data confirmed that methysticin has anti-inflammatory properties as already reported by Pollastri et al. who used an in vitro model of LPS-mediated inflammation in which kavalactones inhibited LPS-induced TNF-α secretion [27]. Recently, we were able to show that pharmacological activation of Nrf2 inhibits NF-κB activity as well as the secretion of the pro-inflammatory cytokines IL-1β and IL-6 in an in vitro model of rheumatoid arthritis [28].

Oxidative damage is a central factor in the pathogenesis of AD [29]. However, clinical trials testing treatments of AD-patients with antioxidants have brought largely negative conclusions [30]. Another way to render neuronal cells more resistant to oxidative stress is to up-regulate the endogenous protection system. Our study show that the oxidative damage caused by amyloid deposition in the brains of APP/Psen1 mice can by significantly reduced by methysticin treatment. Methysticin showed only moderate antioxidant activities [13]. Methysticin functions here as an indirect antioxidant as it can activate the antioxidant, anti-inflammatory and cytoprotective Nrf2-pathway.

## 5 Conclusion

In summary, our data indicate that the prominent long-term memory dysfunction in middle-aged APP/Psen1 mice can be rescued by methysticin treatment that induces the cytoprotective transcription factor Nrf2. Thereby oxidative damage and associated neuroinflammation is significantly reduced. Because of its low side-effect profile and long history (over 3000 years) of safe use, methysticin appears to have considerable potential for clinical application in AD prevention and therapy. Moreover, methysticin may serve as a lead structure in the design of a new class of pharmaceuticals for the treatment of neurodegenerative diseases.

## Competing interests

There are no financial or other relationships that might lead to a conflict of interest.

## Author's contributions

*Athanassios Fragoulis*: study design, methysticin treatment, reporter gene assay, behavioral testing, immunohistochemical stainings against Iba1, GFAP, DT3, 4-HNE and Nrf2, Luminex multi-plex assay, oxyblot, qRT-PCR, microscopy, data acquisition/analysis/interpretation, manuscript draft and revision.

*Stephanie Siegl*: conception of the behavioral tests, data analysis and interpretation, revision of the manuscript.

*Markus Fendt*: data analysis, interpretation and revision of the behavioral testing, revision of the manuscript.

*Sandra Jansen*: immunohistochemical staining against Aβ1-42 and microscopy.

*Ulf Soppa*: Aβ ELISA conduction, data acquisition, revision of the manuscript.

*Lars-Ove Brandenburg*: CongoRed staining and microscopy.

*Thomas Pufe*: study design, critical revision of study and data.

*Joachim Weis*: data analysis and interpretation of immunohistochemical stainings against Iba1 and GFAP.

*Christoph Jan Wruck*: study design, data analysis and interpretation, manuscript draft and revision.
