# Supplementary material for: Oral administration of methysticin improves cognitive deficits in a mouse model of Alzheimer's disease
Source: Redox Biol. 2017 Apr 19;12:843–53. doi: 10.1016/j.redox.2017.04.024 (PMC5406548; doi:10.1016/j.redox.2017.04.024)
Supplement: Supplementary material [file mmc1.docx]

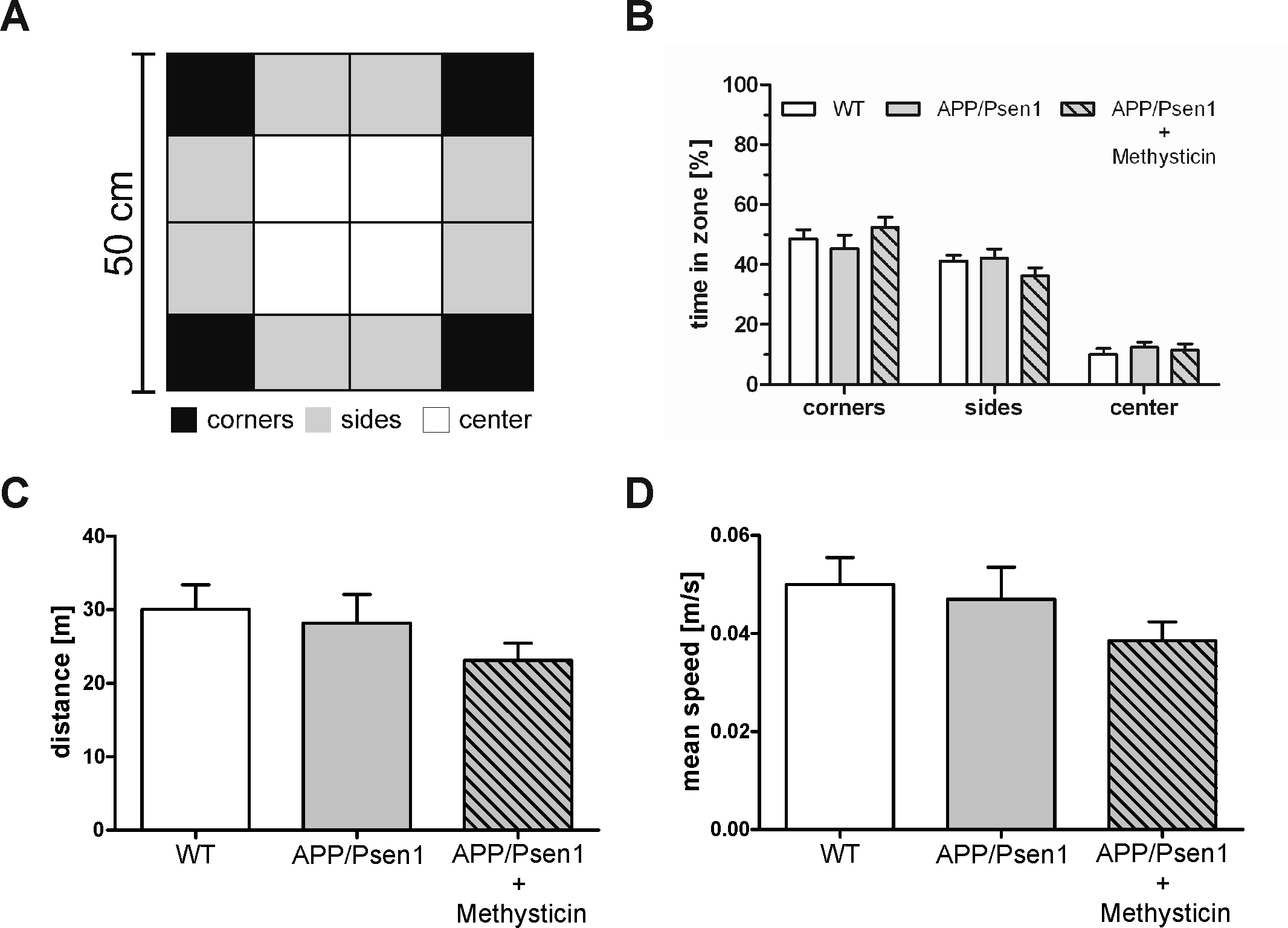


**Fig. S1 Animals’ physical condition is affected neither by APP/Psen1 transgene nor methysticin treatment.** To investigate the animals’ physical condition and species-specific behavior, we used a squared open field apparatus (edge length: 50 cm). The maze was divided into 3 areas: corners, sides, and the center **(A)**. The relative amount of time spent in each zone was determined for each treatment group **(B)**. Furthermore, traveled distance **(C)** and mean speed **(D)** were recorded. There were no differences regarding the physical condition between the investigated groups. (A: two-way ANOVA with Bonferroni post-hoc test; B‑D: one-way ANOVAs with Tukey’s post hoc tests). Data represent mean + SEM; n = 6.


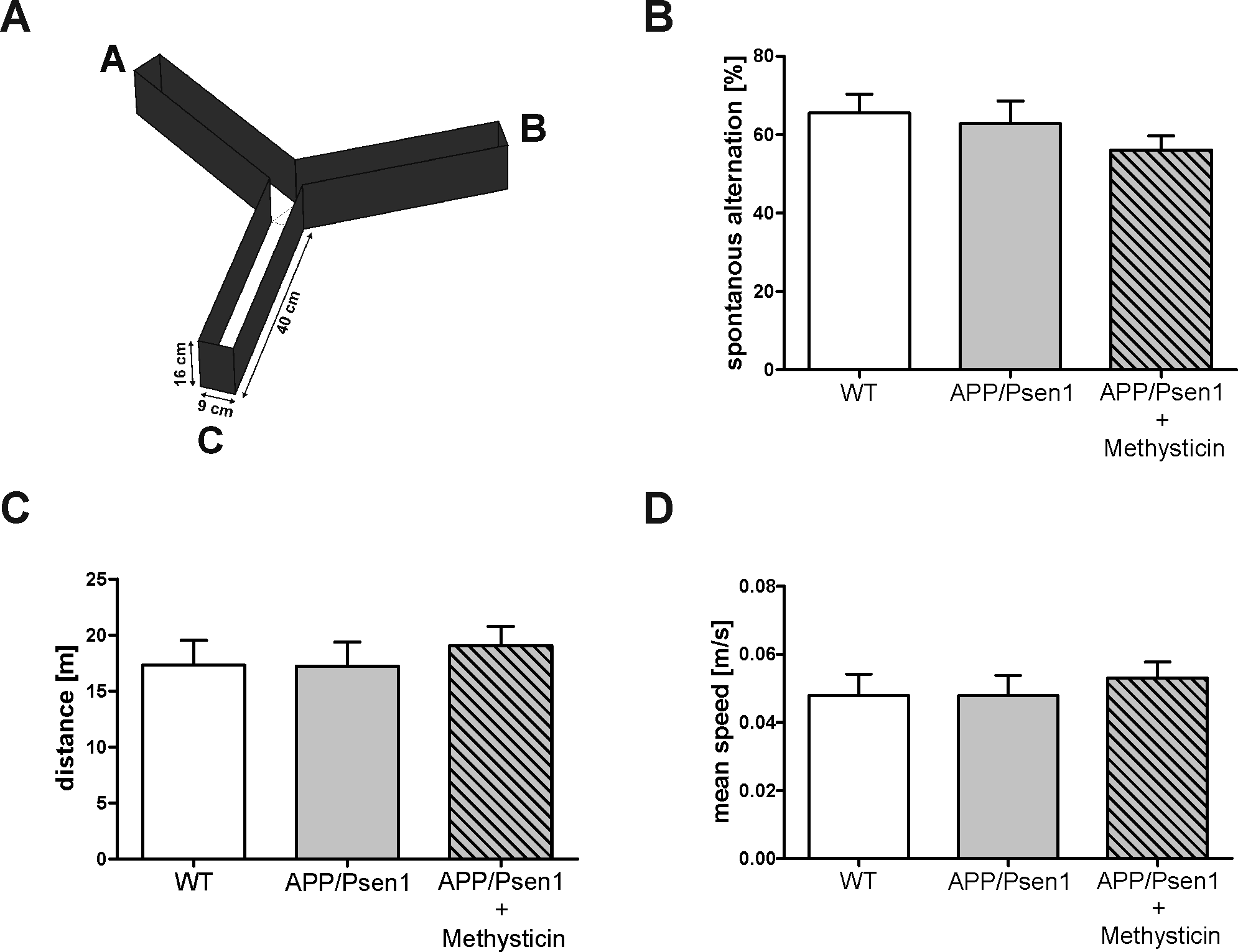


**Fig. S2 Working memory is affected neither by the APP/Psen1 transgene nor by methysticin treatment.** The mice’s working memory was tested in a Y-maze. The maze was composed of three identical arms arrayed at 120° angles to each other. The arms’ dimensions were W x H x D: 9 cm x 16 cm x 40 cm **(A)**. The spontaneous alternation was determined as depicted in equation 1 **(B)**. Furthermore, distances traveled **(C)** and mean speed **(D)** were recorded. There were differences neither regarding the physical condition nor working memory performance between the investigated groups (one-way ANOVAs with Tukey’s post hoc tests). Data represent mean + SEM; n = 6.


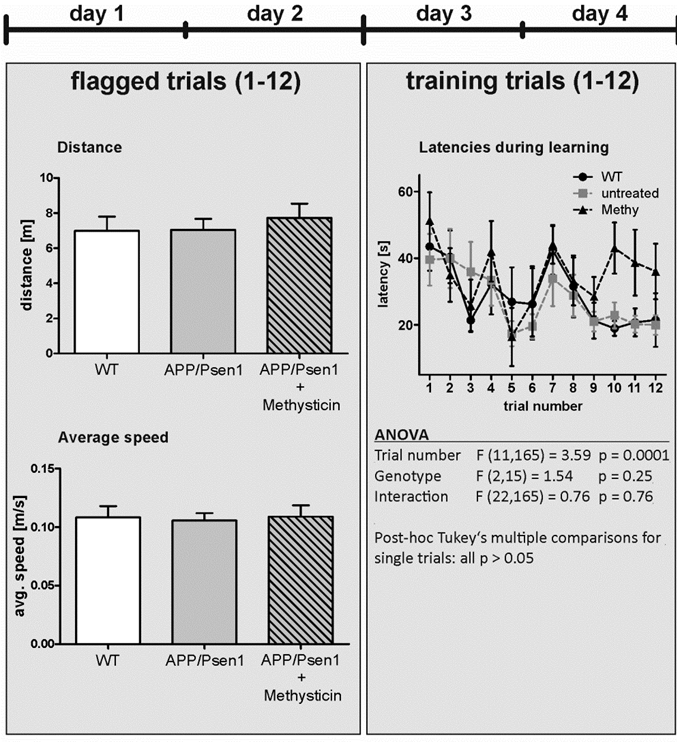


**Fig. S3 Illustration of the Morris-Water Maze flagged and training trials.** To analyze long-term memory we performed a Morris water maze test. The apparatus measured 120 cm in diameter. The overall distance and the average swim speed were quantified during the flagged trials to validate similar locomotor abilities (left panel). The learning process was analyzed during training trials. Learning curves and corresponding statistics are depicted in the right panel. (Statistics flagged trial: one-way ANOVAs with Tukey’s multiple comparisons; statistics training trial: repeated measures two-way ANOVA followed by Tukey’s multiple comparisons for single trials). Data represent mean + SEM; n = 6
